# Modulation of Higher-Order Olfaction Components on Executive Functions in Humans

**DOI:** 10.1371/journal.pone.0130319

**Published:** 2015-06-17

**Authors:** Ana B. Fagundo, Susana Jiménez-Murcia, Cristina Giner-Bartolomé, Mohammed Anisul Islam, Rafael de la Torre, Antoni Pastor, Felipe F. Casanueva, Ana B. Crujeiras, Roser Granero, Rosa Baños, Cristina Botella, Jose M. Fernández-Real, Gema Frühbeck, Javier Gómez-Ambrosi, José M. Menchón, Francisco J. Tinahones, Fernando Fernández-Aranda

**Affiliations:** 1 Department of Psychiatry, University Hospital of Bellvitge-IDIBELL, Barcelona, Spain; 2 CIBER Fisiopatología Obesidad y Nutrición (CIBERObn), Instituto Salud Carlos III, Madrid, Spain; 3 Department of Clinical Sciences, School of Medicine, University of Barcelona, Barcelona, Spain; 4 Integrative Pharmacology and Systems Neuroscience Research Group, Neuroscience Research Program, IMIM (Hospital del Mar Medical Research Institute), Barcelona, Spain; 5 Department of Pharmacology, School of Medicine, Universitat Autònoma de Barcelona, Spain; 6 Endocrine Division, Complejo Hospitalario U. de Santiago, Santiago de Compostela University, Santiago de Compostela, Spain; 7 Departament de Psicobiologia i Metodologia, Universitat Autònoma de Barcelona, Barcelona, Spain; 8 Department of Personality, Evaluation and Psychological Treatment of the University of Valencia, Valencia, Spain; 9 Department of Basic Psychology, Clinic and Psychobiology of the University Jaume I, Castelló, Spain; 10 Department of Diabetes, Endocrinology and Nutrition, Institut d’Investigació Biomèdica de Girona (IdlBGi) Hospital Dr Josep Trueta, Girona, Spain; 11 Metabolic Research Laboratory, Clínica Universidad de Navarra, University of Navarra, IdiSNA, Pamplona, Spain; 12 CIBER Salud Mental (CIBERsam), Instituto Salud Carlos III, Barcelona, Spain; 13 Department of Endocrinology and Nutrition, Hospital Clínico Universitario Virgen de Victoria, Málaga, Spain; Université Lyon, FRANCE

## Abstract

The prefrontal (PFC) and orbitofrontal cortex (OFC) appear to be associated with both executive functions and olfaction. However, there is little data relating olfactory processing and executive functions in humans. The present study aimed at exploring the role of olfaction on executive functioning, making a distinction between primary and more cognitive aspects of olfaction. Three executive tasks of similar difficulty were used. One was used to assess hot executive functions (Iowa Gambling Task-IGT), and two as a measure of cold executive functioning (Stroop Colour and Word Test-SCWT and Wisconsin Card Sorting Test-WCST). Sixty two healthy participants were included: 31 with normosmia and 31 with hyposmia. Olfactory abilities were assessed using the ‘‘Sniffin’ Sticks’’ test and the olfactory threshold, odour discrimination and odour identification measures were obtained. All participants were female, aged between 18 and 60. Results showed that participants with hyposmia displayed worse performance in decision making (IGT; Cohen’s-*d* = 0.91) and cognitive flexibility (WCST; Cohen’s-*d* between 0.54 and 0.68) compared to those with normosmia. Multiple regression adjusted by the covariates participants’ age and education level showed a positive association between odour identification and the cognitive inhibition response (SCWT-interference; Beta = 0.29; *p* = .034). The odour discrimination capacity was not a predictor of the cognitive executive performance. Our results suggest that both hot and cold executive functions seem to be associated with higher-order olfactory functioning in humans. These results robustly support the hypothesis that olfaction and executive measures have a common neural substrate in PFC and OFC, and suggest that olfaction might be a reliable cognitive marker in psychiatric and neurologic disorders.

## Introduction

The olfactory system is directly associated with brain areas involved in cognitive and emotional processes and the evidence of an association between olfactory and some cognitive functions is strong [1–4]. Previous research has suggested a neurocognitive profile illustrating inefficiencies in memory and attentional processing associated with olfactory dysfunction [2,5]. The olfactory alteration in physiological aging, which is usually accompanied by cognitive alterations, is also well documented (hyposmia/anosmia) [1,3,6]. Additionally, and even more importantly from a neuropsychological perspective, the olfactory impairment is a characteristic of certain neurological disorders such as Alzheimer’s (AD) and Parkinson’s (PD) diseases [1,3,6].

The main components of olfaction are odour threshold (OT), odour identification (OI) and odour discrimination (OD) [7]. OT is measured by assessing the lowest concentration of a smell that the person is able to detect. OI and OD show the capacity to identify and differentiate between odorants, respectively. OT is considered to be a more sensorial process and mainly depends on the peripheral and subcortical part of the olfactory system. On the other hand, OD and mainly OI are more cognitive tasks and are mediated by cortical-subcortical circuits [8]. It has been well established that these three components are impaired in neurodegenerative disorders, such as AD or PD [1,3,6]. However, OI is impaired from the early stages of the disorder, similar to cognitive decline, while OT alterations appear at later stages [9]. Interestingly, impairment of OI in AD is a predictor of memory loss and is correlated with the Mini Mental State [9].

However, the relation between olfaction with more complex cognitive functions such as executive functions has not been properly addressed, despite the overlap between their brain circuits. Executive functions (EF) are higher order cognitive capacities that allow people to orient towards the future, display self-control and effectively have goal oriented behaviour [10]. EF can be split into two main categories: ‘cold’ EF, which includes the more rational or logical processes, and ‘hot’ EF which is more related to emotional or motivational processes [11]. The neural substrate of EF is the prefrontal cortex (PFC) [12]. The PFC is divided into two subregions: medial prefrontal cortex (mPFC) (i.e. anterior cingulate cortex or prelimbic cortex) and orbitofrontal cortex (OFC) (i.e. dorsolateral or ventralorbital cortex) [12,13]. Consistent with this neuroanatomical division, different areas of PFC are implicated in the performance of particular executive functions. Specifically, the mPFC is responsible for cognitive flexibility involving set shifting [14,15] while the OFC controls decision making [16]. As for the association between executive functions and olfactory processing, few studies were conducted. They mainly focused on elderly individuals, patients with amnestic mild cognitive impairment, early Alzheimer's disease or Parkinson’s disease [17–19]. Although these are interesting results demonstrating impaired olfactory functioning to be an early marker of medial temporal lobe degeneration, the impact of the neurodegeneration ought to be considered.

Nevertheless, and in spite of this interesting hypothesis, there are no studies linking the different components of olfaction and EF in healthy young population, demonstrating the physiological role of olfaction on higher cognitive functions in humans. This study aimed to assess the association between olfactory functioning and executive functions while evaluating hot and cold executive functions in healthy subjects with hyposmia and normosmia. We made a distinction between primary and more cognitive aspects of olfaction and expected an association between executive processing and the higher-order components of olfaction, mainly the identification capacity.

## Methods

### Sample

All participants were informed about the research procedures and gave informed consent in writing. Procedures were approved by the Ethical Committee of each of the following institutions: University Hospital of Bellvitge-IDIBELL; University Hospital of Santiago (Santiago de Compostela); Clinic University Hospital Virgen de Victoria, Málaga; University of Navarra, Pamplona; Biomedical Research Institute of Girona (IdIBGi-Doctor Josep Trueta Hospital, Girona); IMIM (Hospital del Mar Medical Research Institute, Barcelona; University Jaume I, Castelló.

Seven centers, all involved in the CIBERobn Spanish Research Network, participated. They include: the Eating Disorders Unit (Department of Psychiatry, University Hospital of Bellvitge-IDIBELL, Barcelona), the Department of Endocrinology at the University Hospital of Santiago (Santiago de Compostela); the Department of Diabetes, Endocrinology and Nutrition (Clinic University Hospital Virgen de Victoria, Málaga); the Department of Endocrinology and Nutrition (University of Navarra, Pamplona); the Diabetes, Endocrinology and Nutrition Department, Biomedical Research Institute of Girona (IdIBGi-Doctor Josep Trueta Hospital, Girona); the Human Pharmacology and Clinical Neurosciences Research Group at IMIM (Hospital del Mar Medical Research Institute, Barcelona) and the Department of Basic Psychology, Clinic and Psychobiology (University Jaume I, Castelló).

Sixty two consecutive healthy subjects (n = 62) were included. 31 had normosmia and 31 had hyposmia that was detected in the TDI-score of the Sniffin’ Sticks [20]. All participants were female, aged between 18 and 60 and spoke Spanish as their mother tongue. Participants were recruited through several sources including word-of-mouth and advertisements in the local university. Sociodemographic characteristics, as well as the distribution of some clinical variables (use of oral contraceptives, tobacco use and olfactory measures), are presented in Table 1.

**Table 1:** Descriptives for sample.

	Normosmia (n = 31)	Hyposmia(n = 31)	^*1*^ *Statistic*	*df*	*p*
Age (years); *mean (SD)*	26.39 (8.45)	25.42 (8.57)	t = 0.448	60	.656
Civil status; *%*			χ^2^ = 3.093	2	.213
*Single*	63.3%	71.0%			
*Married—in couple*	36.7%	22.6%			
*Divorced—separated*	0%	6.5%			
Employment status; *% Employed*	54.8%	35.5%	χ ^2^ = 2.345	1	.126
Education level; *%*			χ ^2^ = 0.848	2	.654
*Primary*	6.5%	12.9%			
*Secondary*	54.8%	54.8%			
*University*	38.7%	32.3%			
Years of education; *mean (SD)*	16.84 (2.92)	15.65 (2.54)	t = 1.717	60	.091
Use of oral contraceptives; *%*	29.0%	12.9%	χ ^2^ = 2.433	1	.119
Smoker; *%*	25.8%	32.3%	χ ^2^ = 0.313	1	.576
Number of cigarettes-day; *mean (SD)*	2.61 (5.36)	2.32 (4.61)	t = 0.229	60	.820
***Olfactory measures***					
*Discrimination; mean (SD)*	13.55 (1.39)	11.35 (1.76)	t = 5.448	60	< .001
*Identification; mean (SD)*	13.71 (1.42)	11.26 (1.55)	t = 6.499	60	< .001
*Threshold test (TDI); mean (SD)*	34.57 (2.24)	27.77 (2.62)	t = 10.98	60	< .001

Results are presented as Mean (Standard deviation). TDI: threshold test

The exclusion criteria were: (1) History of chronic medical illness or neurological condition that might affect cognitive function and/or olfactory functioning; (2) Head trauma with loss of consciousness for more than 2 min, intellectual disability; (3) Use of psychoactive medication or drugs (4) Being male; (5) Age under 18 or over 60 (to discard neuropsychological deficits associated with age); (6) Having diabetes type I or II. The lifetime history of health or mental illnesses profile was based on the general health questionnaire GHQ-28[21].

### Assessment

#### Neuropsychological assessment

As described in previous studies [22,23], all participants underwent a comprehensive neuropsychological and clinical assessment. The neuropsychological tests were selected to cover various aspects of both cold and hot executive functions including decision making, response inhibition, strategic planning and cognitive flexibility. They were administered by a trained psychologist in a single session and in a randomized order. All participants were assessed with the following neuropsychological tests:

Wisconsin Card Sorting Test [24]: This is a classical measure of planning capacity, cognitive flexibility, capacity of shifting among stimulus, and control of impulsive responses not aimed at achieving an objective. Subjects have to match a target card with one of four category cards: a single red triangle, two green stars, three yellow crosses, and 4 blue circles. Cards might be matched by color, number or shape. After each trial a feedback is given to the participant, indicating if they have matched the card correctly. However, during the task the classification rule is unpredictably changing. The test ends when the participant has completed 6 categories or 128 trials.

Stroop Color and Word Test [25]: This paper and pencil test has shown adequate reliability and construct validity for the assessment of inhibition and switching skills. The SCWT measures interference control, flexibility and attention. The task included three pages: (1) a page with color words printed in black ink; (2) a page with “Xs” printed in three different colors; (3) a page with names of colors printed in an incongruent color (i.e. word “blue” printed in red ink). Participants have 45 seconds to read as many words as possible in the first page and name the ink color in pages 2 and 3. Three scores are obtained after task completion: number of words (page 1), number of color-named “X” (page 2) and number of color-named words (page 3). An additional “interference score” is obtained. Higher scores in this variable indicate a better capacity of inhibition response.

Iowa Gambling Task [26]: This computer task evaluates decision-making, risk and reward and punishment value. The subject has to select 100 cards from four decks (A, B, C and D). After each card selection an output is given: gain or a gain and loss of money. Two decks (A and B) are not advantageous as the final loss is higher than the final gain. Decks C and D, however, are advantageous since the punishments are smaller. The final objective of the task is to make the most profit and win as much money as possible. This test is scored by subtracting the amount of cards selected from decks A and B from the amount of cards selected from decks C and D. Higher results point to better performance while negative results point to preference for the not advantageous decks.

#### Olfactory assessment

OT, OD and OI were investigated using the Sniffin’ Sticks [20]. A trained researcher carried out the tests in the following order: (a) olfactory threshold; (b) olfactory discrimination; and (c) olfactory identification. The subjects wear blindfolds during the olfactory assessment.

Olfactory threshold test: Three pens were presented in a randomized order, two contained odourless samples and the third contained an odorant sample. The task of the subject was to indicate the pen with the odorant. Concentration was augmented if the subject chose an odourless pen and reduced if the correct pen was recognized twice. A total of 16 odour concentrations were tested. The mean of the last four of seven trials was used, ranging from 1 to 16. The higher the score the higher the olfactory capacity.

Olfactory discrimination test: The subjects were asked to discriminate between 16 triplets of odours. In each group 2 odours were identical and 1 odour was different and the task was to recognize the odour that was different. The total score was the sum of correct responses ranging from 0 to 16. Higher scores were indicative of a better discrimination capacity.

Olfactory identification test: A pen with an odour was presented to the subject. The pens contain common fragrances, such as peppermint, orange, leather, cinnamon, banana, garlic, lemon, rose, coffee, apple, clove, pineapple, aniseed and fish. Subjects were asked to identify the odour, by choosing it in a card listing four terms, only one of which correctly identified the odour. For this purpose the eye mask was removed (only for reading the card). The total score ranged from 0 to 16, with higher scores indicating a better identification capacity.

TDI-score: The sum of the scores from the three subtests resulted in the TDI-score (Threshold, Discrimination, and Identification) with a maximum of 48 points. As defined in [27], a score of 30.5 points or more indicates normosmia, a score between 16.5 and 30 points indicates reduced olfactory function in terms of hyposmia, and a score of less than 16.5 points indicates an olfactory functional impairment or anosmia.

### Statistical analysis

Analyses were carried out with SPSS20 for Windows. First, T-TEST proceduresd compared the mean scores of the cognitive measures between participants into the normosmia and hyposmia groups. To control the potential inflation of Type-I error due to multiple statistical comparisons (one for each cognition measure) and since Bonferroni's correction method has largely been criticized for being too conservative, an alternative procedure was considered: Simes’ procedure [28], an improved modified method for the test of an overall hypothesis which is the combination of k individual hypothesis with the advantage to be more powerful over the classical Bonferroni system and particularly advantageous when several highly-correlated test statistics are involved. Additionally, since p-values are strongly dependent to sample sizes and that from a practical-clinical perspective to estimate the effect size is the main objective of the empirical research. In this study each mean difference included its effect size through Cohen’s-d coefficient (moderate effect size was considered for |d|>0.50 and high effect size for |d|>0.80).

Next, multiple regression analyses valued the contribution of olfactory scores on the cognitive measures. These regressions were modelled in two steps: first step entered and fixed the covariates participants’ age and years of education, and second step added the independent variables odour discrimination and odour identification.

## Results

**Fig 1:**
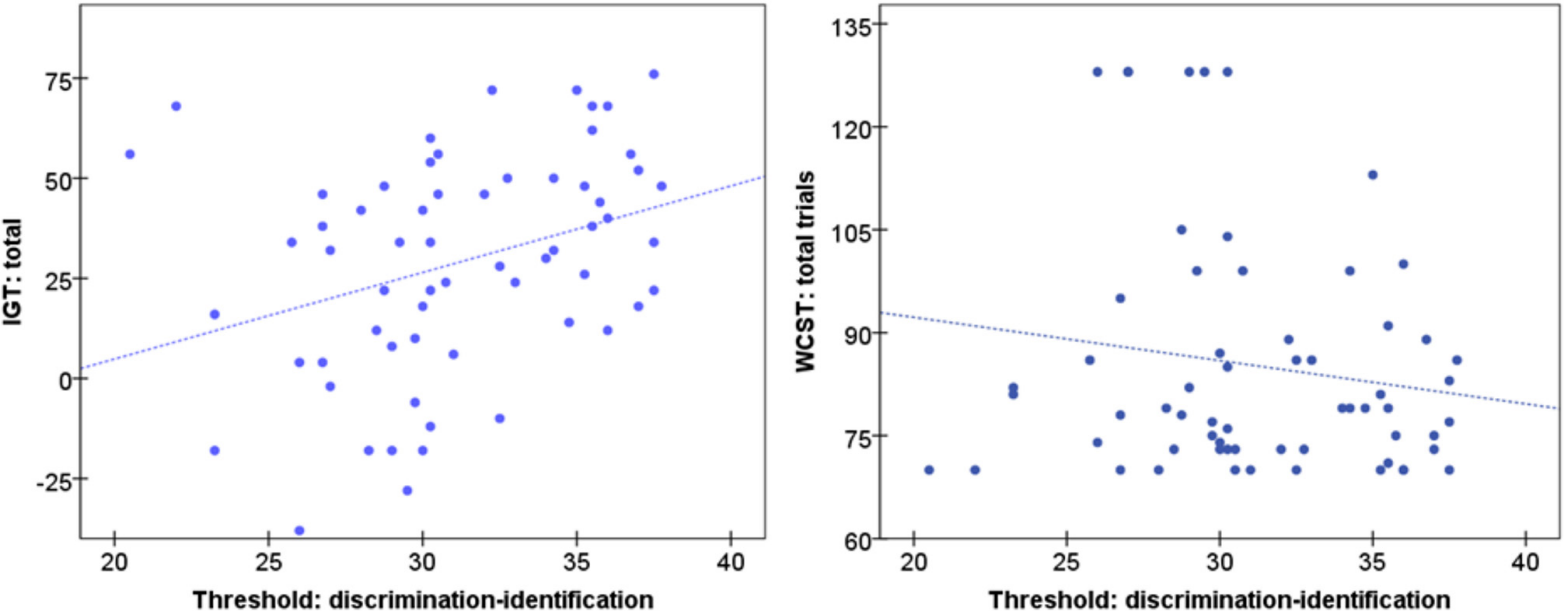
Scatter-plots for the odor threshold discrimination-identification score and the IGT-total and the WCST-total trials (dash-line represents the lineal total adjustment).

Table 2 shows the results of the T-TEST comparing the mean cognitive scores between participants into the normosmia and hyposmia groups, as well as the effect size of each mean difference. As a whole, these results indicate that hyposmic participants displayed worse performance in decision making and cognitive flexibility: many mean differences achieved significant results and its effect size was within the moderate to high range. Specifically, the IGT decision making mean score was significantly lower for hyposmic than normosmic subjects (first scatter-plot in Fig 1 shows the graph of the threshold for discrimination-identification score and IGT total). The hyposmia group also registered a mean performance on the WCST scales significantly worse than that of normosmia group (except for the factor total number of corrects) (second scatter-plot in Fig 1 shows the graph of the threshold for discrimination-identification score and the WCST-total trials). No significant differences on inhibition response performance (STROOP) appeared between the normosmic and hyposmic conditions.

**Table 2:** Comparison of cognitive scores between normosmia and hyposmia.

	Normosmia		Hyposmia		Mean comparison: T-TEST				
	*n* = 31		*n* = 31		*T-stat*	Sig.	Mean	95% CI	Cohen’s
	Mean	SD	Mean	SD	*(df = 60)*	^*1*^ *p*	differ.	for mean differ.	*| d |*
***SCWT***									
Interference	5.03	9.69	4.11	6.91	0.430	.669	0.92	-3.38; 5.22	0.11
***WCST***									
Total trials	80.58	11.02	89.81	21.16	2.154	**.050**	-9.23	-17.80; -0.66	0.55*
Total corrects	67.77	5.04	64.87	9.06	1.541	.125	2.91	-0.83; 6.65	0.40
Total errors	12.81	6.76	24.68	25.60	2.496	**.036**	-11.87	-21.38; -2.36	0.63*
Perseverative responses	7.35	4.58	12.39	12.86	2.052	**.050**	-5.03	-9.94; -0.13	0.52*
Perseverative errors	7.00	4.01	11.55	11.32	2.109	**.050**	-4.55	-8.86; -0.23	0.54*
Non-persev. Errors	5.81	3.48	13.13	15.32	2.596	**.036**	-7.32	-12.97; -1.68	0.66*
Total categories completed	6.00	0.00	4.93	2.23	2.667	**.036**	1.07	0.27; 1.87	0.68*
Trials to first category	11.45	1.15	29.90	42.12	2.439	**.036**	-18.45	-33.59; -3.31	0.62*
***IGT***									
Total	40.39	21.17	17.61	28.37	3.583	**.001**	22.77	10.06; 35.49	0.91*

1p-value includes Simes’ correction procedure for multiple comparisons. SD: standard deviation. Bold: significant comparison (.05 level).

*Moderate to high effect size (|d|≥0.50).

Table 3 contains the results of the second-step of the multiple regressions valuing the predictive capacity of olfactory capacity (measured through the odour discrimination and the odor identification scores) on the cognitive measures (the first step of the regressions entered and fixed the covariates participants’ age and years of education). Odour identification was significantly associated with the SWCT interference measure: higher capacity of identification predicted better performance on this cognitive measure. Odour identification was not associated with the cognitive flexibility capacity (WCST) or with the IGT executive performance score. The odour discrimination capacity was not a statistical predictor of the olfactory levels.

**Table 3:** Predictive capacity of global olfactory functions on cognitive scores: beta-coefficients in multiple linear regressions.

	Odor discrimination			Odor identification			^1^Model change		
	*Beta*	*T-stat*	*p*	*Beta*	*T-stat*	*p*	*F*_*(2;57)*_	*p*	*R* ^*2*^
***SCWT***									
Interference	-0.048	-0.364	.717	**0.287**	2.171	**.034**	2.478	.093	.073
***WCST***									
Total trials	-0.148	-1.162	.250	-0.021	-0.162	.872	0.900	.412	.025
Total corrects	0.092	0.648	.520	0.146	1.018	.313	1.177	.316	.040
Total errors	-0.160	-1.252	.216	-0.078	-0.603	.549	1.489	.234	.041
Perseverative responses	-0.140	-1.163	.250	-0.047	-0.391	.697	1.100	.340	.027
Perseverative errors	-0.155	-1.278	.206	-0.038	-0.312	.756	1.207	.307	.030
Non-persev. Errors	-0.153	-1.145	.257	-0.102	-0.759	.451	1.515	.228	.046
Total categories completed	0.145	1.073	.288	0.087	0.640	.525	1.252	.294	.038
Trials to first category	-0.171	-1.246	.218	-0.062	-0.450	.655	1.308	.278	.041
***IGT***									
Total	0.039	0.333	.740	**0.218**	1.876	.066	2.433	.097	.055

Bold: significant parameter (.05 level). 1Model change comparing first step (including the covariates age and education) and second step (adding the odor discrimination and odor identification scores).

## Discussion

This study set out to examine the association between different components of olfaction and executive functions in humans. A significant association was observed between higher-order component of olfaction (identification) and cognitive flexibility, inhibition response and decision making. Our results suggested that subjects with normosmia showed a better capacity of set shifting and less sensibility to immediate reward (IGT) compared with subjects with hyposmia. Although olfactory modulation was previously related to cognitive performance in humans [1–4], this is, to the best of our knowledge, the first study to explicitly test the hypothesis of altered interactions between cognitive components of olfaction and executive control networks among healthy individuals.

The main result of our study is the differentiation between the different components of olfaction on their effects on the executive functioning. According to our results only the identification component is linked to inhibition response cognitive flexibility and decision making. These results are in line with those showing that the ability to identify odours is linked to prefrontal, particularly OFC functioning [29], brain regions associated with executive functioning. This hypothesis has also been supported by early patient lesion studies implicating OFC regions in odour identification [30,31]. Our results also support the hypothesis that other components of olfaction, such as the olfactory threshold, are less cognitive and more associated with sensorial processing [8,9].

Specifically, the association between the odour identification capacity and the IGT performance, found by means of the regression analysis, is in line with the hypothesis of the OFC implication in both processes. The IGT is a classic neuropsychological task for measuring the capacity of making decision and the sensibility to immediate reward. Different profiles in IGT performance were found between groups: Subjects with normosmia performed better and learned to keep away from not advantageous decks, while the performance of hyposmia participants did not improve along the task. This group went for choices that result in elevated immediate gains despite important future losses. Thus, subjects with lower olfactory capacity have an incapacity to successfully regulate reward and punishment, which might be translated into deficit in planning capacity. The cognitive mechanism underlying the decision making performance in these subjects might be associated with an elevated level of impulsivity. It has been established that impulsive subjects have marked limitations for learning suitable associations between reward and punishment [32,33]. As a consequence, there is a tendency for these subjects to have a reduced capacity to delay gratification, showing a reward based impulsivity. Overall, this cognitive profile is particularly important as a cognitive marker in psychiatric and neurologic disorders considering that it might be an expression of difficulties to successfully regulate reward and punishment, which might be translated into deficit in planning every day functioning.

Our results are also in line with neuroimaging studies supporting the hypothesis of the PFC and OFC as common neural substrates of olfaction and executive functioning, although a direct association cannot be addressed considering that we did not use neuroimaging techniques [34,35]. Taken as a whole, functional magnetic resonance imaging (fMRI) studies demonstrate an odour-evoked neural activity in the left OFC during presentation of odour stimuli, demonstrating the critical role of this area in human olfactory consciousness [35]. In the same line, several studies have demonstrated odour-evoked OFC activation and recent fMRI studies have robustly associated the OFC with odour identity [36–38]. Particularly relevant for the decision making capacity are those studies that demonstrated the differential patterns of fMRI (or positron emission tomography) activity in human OFC induced by pleasant and unpleasant odours [39–41]. These studies indicate that the OFC encodes representations not only of odour identity, but also odour valence and acquired olfactory value, which is finally translated into food or odour-related decisions. As for the IGT, several studies have demonstrated the role of OFC in its performance. The IGT simulates daily-life decision-making [42] and healthy subjects usually learn to choose cards from the advantageous decks instead of persisting on choosing the disadvantageous options. Conversely, patients with focal cerebral lesions in the PFC and OFC are unable to acquire this affective learning and remain choosing the disadvantageous decks still after significant losses [43–45], corroborating the role of these areas in the effective decision making capacity.

Our study has several important strengths including the relatively large sample size of healthy young subjects with hyposmia. Additionally, we have used a standardized method for testing the olfactory capacity differentiating between three components of olfaction. Most of the previous studies on the topic had been conducted in psychiatric or neurologic populations using cognitive tasks that measure memory or attention. Conversely, our study was specifically designed to test the executive functioning, by using three well validated executive tests.

However, the results of this study should be interpreted within the context of some limitations. First, only females were included in the study, thus the results are not applicable to males. Further studies including both males and females should be desirable. Second, measures of intelligence quotient, which is an important variable to take into account in cognitive studies, were not included. Nevertheless, as a cognitive level measure, years of education, has been considered, and no significant differences were observed between experimental groups. Third, neuroimaging data was not collected, thus future studies employing these techniques are desirable in order to confirm a common neural substrate for olfactory and executive functioning. Finally, future studies should also consider including additional executive tasks (decision-making, inhibition response and cognitive flexibility) in order to shed more light on their interactions and possible brain mechanisms.

To summarize, this study demonstrates that in humans, the olfactory capacity plays an important role on prefrontal-dependent cognitive functions, almost certainly by common cerebral circuits. This study might have implications in the research of the executive profile of psychiatric and neurologic disorders, including abnormal eating behaviours [22] and schizophrenia [1], given the recent evidence on the role of olfaction on these disorders. According to our results, patients with predispositions associated with poorer identification capacity are at a higher risk of presenting difficulties with decision making and cognitive flexibility. Understanding the mechanisms involved in this profile may contribute to the development of new treatments and pharmacological approaches for these disorders.
